# Risk and Burden of Preterm Birth Associated with Prenatal Exposure to Ambient PM_2.5_: National Birth Cohort Analysis in the Iranian Population

**DOI:** 10.3390/toxics13080680

**Published:** 2025-08-15

**Authors:** Ling Tong, Yalin Zhang, Yang Yuan, Fatemeh Mayvaneh, Yunquan Zhang

**Affiliations:** 1 School of Health and Nursing, Wuchang University of Technology, Wuhan 430223, China; 2 School of Public Health, Wuhan University of Science and Technology, Wuhan 430065, China; 3 Shenzhen Bao’an District Songgang People’s Hospital, Shenzhen 518100, China; 4 Institute of Epidemiology and Social Medicine, University of Münster, 48149 Münster, Germany; 5 Faculty of Geography and Environmental Sciences, Hakim Sabzevari University, Sabzevar 9617916487, Iran

**Keywords:** fine particulate matter, air pollution, preterm birth, disease burden, Iran

## Abstract

Preterm birth (PTB) is a major global public health concern with substantial impacts on neonatal morbidity and mortality. There is a growing body of evidence linking maternal exposure to fine particulate matter (PM_2.5_) with PTB, and national birth cohort data from the Middle East remains sparse. We analyzed 3,839,531 singleton live births in Iran from 2013 to 2018. Monthly PM_2.5_ concentrations during pregnancy were estimated using validated spatiotemporal models. Associations between prenatal PM_2.5_ exposure and multiple PTB subtypes, moderate to late (MPTB), very (VPTB), and extremely preterm birth (EPTB), were assessed using multivariable logistic regression. A 10 μg/m^3^ increase in PM_2.5_ was associated with increased odds of PTB (odds ratio [OR] = 1.048, 95% confidence interval [CI]: 1.044–1.051), MPTB (OR = 1.046, 95% CI: 1.042–1.049), VPTB (OR = 1.059, 95% CI: 1.048–1.070), and EPTB (OR = 1.064, 95% CI: 1.047–1.081), respectively. Age- and trimester-stratified analyses showed greater exposure-related risks among mothers aged 25–34 and during mid-pregnancy. We observed consistent evidence for a J-shaped exposure–risk pattern in overall and subgroup populations, suggesting a PM_2.5_ threshold near 40 μg/m^3^. From 2013 to 2018, 6716 (95% CI: 5336–8678) PTB cases, representing 2.7% (95% CI: 2.2–3.5%) of total PTB, were attributable to PM_2.5_ exposure exceeding the WHO first-stage interim target (IT1, 35 μg/m^3^). Our results suggested improved ambient PM_2.5_ quality may substantially reduce PTB burden in Iran.

## 1. Introduction

Preterm birth (PTB) is a crucial global public health issue and a leading cause of under-5 mortality [1]. Preterm infants are usually faced with greater risks of adverse health outcomes throughout life, including intellectual and psychiatric impairment in childhood and early onset of chronic diseases in adulthood [2]. According to the report released by the World Health Organization (WHO), 13.4 million preterm infants were born globally in 2020, nearly representing one-tenth of all live births [3]. Notably, more than 90% of PTB cases occurred in the regions of low- and middle-income countries (LMICs) [4], where the vast majority of the population are suffering from poor ambient air quality [5].

Maternal exposure to air pollution during pregnancy, particularly fine particulate matter (PM_2.5_), has been associated with PTB outcomes in Europe [6], North America [7], and East Asia [8]. However, national birth cohort evidence from many LMICs is lacking [9,10], especially in Middle Eastern countries, and combined with environmental features, such as arid climate, frequent dust storms, and high ambient PM_2.5_ levels, findings differ markedly from those of high-income regions [11]. Prior global assessments utilized unified exposure–risk associations to quantify PM_2.5_-related PTB burden [12], which may introduce great uncertainty in Middle Eastern populations due to potentially heightened population vulnerability to environmental hazards. Moreover, population-based evidence on the exposure–response (E-R) relationship between air pollution and PTB remains scarce in this region, limiting the understanding of the potential benefits of air pollution reduction on PTB burden.

In this study, we aimed to estimate the associations between PM_2.5_ exposure and various PTB outcomes using a nationwide registry-based data of approximately 4 million birth records in Iran. We developed E-R curves for age-specific mother groups to explore the potential nonlinear associations and quantified the avoidable burden of PTB attributed to ambient PM_2.5_ under the counterfactual scenario of improved air quality.

## 2. Methods

### 2.1. Data Collection

We obtained data from the Iranian Ministry of Health and Medical Education, a nationally representative delivery network across 31 provinces in Iran [13]. A total of 4,068,843 birth records were initially extracted from 749 hospitals, spanning from January 2013 to March 2018. This study was ethically approved by the Ethics Committee of Sabzevar University of Medical Sciences (IR.MEDSAB.REC.1396.99). A total of 5.6% (*n* = 229,312) of the initial records were excluded due to maternal age beyond the WHO-defined fertile range (15–49 years) and incomplete demographic characteristics and unmatched exposure. The final analytical sample included 3,839,531 singleton live births with determined infant sex. We collected information on samples including fetal variables, maternal demographic and health status, and the address of the delivery hospital. As recommended by WHO, PTB was defined as a gestational age (GA) of 20–<37 weeks (from 20 weeks up to, but not including, 37 completed weeks) [3], which was typically calculated by weeks from the first day of the last menses to the birth date. In line with prior investigations [14,15], the overall PTB was categorized into moderate to late PTB (MPTB, 32–36 weeks), very PTB (VPTB, 28–31 weeks), and extremely PTB (EPTB, 20–27 weeks).

Monthly surface PM_2.5_ concentrations at a spatial resolution of 0.1° × 0.1° across the entire Iran were derived from the V5.GL.02 dataset developed by the Atmospheric Composition Analysis Group at Washington University. Information from the GEOS-Chem chemical transport model, satellite observations, and ground-based monitoring stations was integrated to estimate spatiotemporal PM_2.5_ concentrations globally and regionally [16,17], which has been widely used in large-scale environmental health investigations in the past decade. Due to the paucity of maternal residential addresses, we matched PM_2.5_ exposure during pregnancy according to the delivery hospital address of each mother-baby pair. This alternative approach has also been widely used in prior large-scale epidemiological studies where residential information of the population was unavailable [18,19] and is particularly reasonable in contexts like Iran, where most women deliver at hospitals close to their homes due to the structure of the healthcare system [20]. To investigate the effects of PM_2.5_ on PTB at each stage of pregnancy, the exposure was separately assessed for multiple windows: the first trimester (1–13 weeks of gestation), the second trimester (14–26 weeks of gestation), the third trimester (27 weeks to delivery), and the entire pregnancy.

### 2.2. Data Analysis

The characteristics of the sample were summarized as mean (standard deviation, SD) for continuous variables and frequencies (proportions) for categorical ones. A two-stage approach was utilized in the analyses. In the first stage, we investigated the associations between PM_2.5_ exposure and PTB outcomes using multivariable-adjusted logistic regression models. In the second stage, we estimated the avoidable burden of PTB attributable to PM_2.5_ exposure exceeding a predefined target, on the basis of the potential nonlinear E-R association. All data analyses were conducted in R software version 4.3.0 (R Foundation for Statistical Computing, Vienna, Austria). A two-sided *p*-value of less than 0.05 was considered statistically significant.

#### 2.2.1. Associations of PM_2.5_ Exposure with PTB Outcomes

Logistic regression models adjusted for multiple fetal and maternal covariates were utilized to evaluate the associations between PM_2.5_ exposure and PTB outcomes [13,21,22]. Fetal variables included sex (male or female), the season of birth (spring, summer, autumn, or winter), and delivery type (cesarean section or vaginal delivery); and maternal variables included age (15 to 24 years, 25 to 29 years, 30 to 34 years, or 35 to 49 years), education attainment (below high school, high school, or college and above), residence (city or village), nationality (Iranian or non-Iranian), pre-pregnancy status of diabetes (presence or absence) and hypertension (presence or absence), parity (primipara or multipara), and history of abortion (presence or absence). Age was grouped as 15–24 years, 25–29 years, 30–34 years, and 35–49 years based on clinical relevance and epidemiological conventions, with distinctions for adolescent pregnancies [23], variations within reproductive age [24], and advanced maternal age [25]. We estimated the odds ratio (OR) and corresponding 95% confidence interval (CI) associated with a 10 μg/m^3^ increase in PM_2.5_ during each exposure window. Sensitivity analyses were further conducted by sequentially adjusting for area-level socioeconomic indicators, including medical insurance participation (MIP) and unemployment rate (UR) in the analytic models. These area-level socioeconomic indicators were sourced from the National Statistical Yearbook of Iran [21,22]. Two-sample z-tests were used to compare the potential effect differences between models with various adjustment strategies.

To identify the risk susceptibility of various maternal age groups, we performed age-stratified analyses for PM_2.5_-PTB associations and used a fixed-effects meta-regression method to detect any heterogeneity in the effects across different age cohorts [26]. We explored the potential nonlinear E-R relationships using a restricted cubic spline (RCS) term with three knots placed at the 10th, 50th, and 90th percentiles of PM_2.5_ exposure distribution, a flexible and commonly used approach in air pollution epidemiology [13,27]. Model fit was also evaluated using the Akaike Information Criterion (AIC) to compare overall model adequacy. The adequacy of fit between linear and RCS models was assessed via the likelihood-ratio test, with a *p*-value < 0.05 being indicative of significant deviation from the linearity assumption [27].

#### 2.2.2. Assessment of PTB Burden Attributable to PM_2.5_

We quantified the burden of PTB attributed to PM_2.5_ from 2013 and 2018, utilizing a counterfactual analysis framework that has been widely employed in previous studies [12,24]. Based on observed age-specific E-R functions, we first estimated the exposure-related odds of PTB for given PM_2.5_ concentrations at the hospital level, using the WHO first-stage interim target (IT1, 35 μg/m^3^) as the counterfactual exposure. This choice of counterfactual exposure was in accordance with the achievable goal under the current pollution context in Iran during 2013–2018 and our E-R functions suggesting a potential threshold around 40 μg/m^3^. OR estimates were then converted to relative risk (RR) by applying Equation (1) to avoid potential overestimation [28]. The national-level attributable number (AN) of PTB cases was finally determined by aggregating the AN estimate across 749 hospitals in Equation (2). Hospital-specific AN was obtained by multiplying its registered number of PTB by the attributable fraction (AF), which was computed using the estimated RR for PTB in each hospital. We employed Equation (3) to derive an overall AF of PM_2.5_-related PTB, dividing the national estimated AN by the total number of PTB cases recorded across all hospitals.

(1) $$RR_{h,g}=\frac{OR_{h,g}}{\left(1-P_{g}\right)+P_{g}\times OR_{h,g}}$$

(2) $$AN_{g}=\sum_{h}N_{h,g}\times \frac{RR_{h,\;g}-1}{RR_{h},g}$$

(3) $$AF_{g}=\frac{AN_{g}}{\sum_{h}N_{h,g}}\times 100\%$$

In Equations (1)–(3), *h* denotes the hospital units (1–749), and *g* represents the maternal age group (e.g., 15 to 24 years, 25 to 29 years, 30 to 34 years, or 35 to 49 years); *P_g_* refers to the prevalence of PTB among maternal age group *g*, which was gained from our national delivery dataset between 2013 and 2018; *N_h,g_* signifies the registered number of PTB cases from hospital *h* for maternal age group *g*; *OR_h,g_* represents the OR estimates attained from E-R functions for maternal age group *g*, taking IT1 (annual 35 μg/m^3^) as a reference exposure for infants born in hospital *h. OR_h,g_* is set as 1 for a given hospital *h* with an annual PM_2.5_ concentration meeting WHO IT1 (≤35 μg/m^3^), indicating that there is no exposure-related excess risk and attributable PTB cases. The total AN was calculated by summing the AN of each maternal age group, considering the potential effect of heterogeneity between groups.

## 3. Results

**Figure 1:**
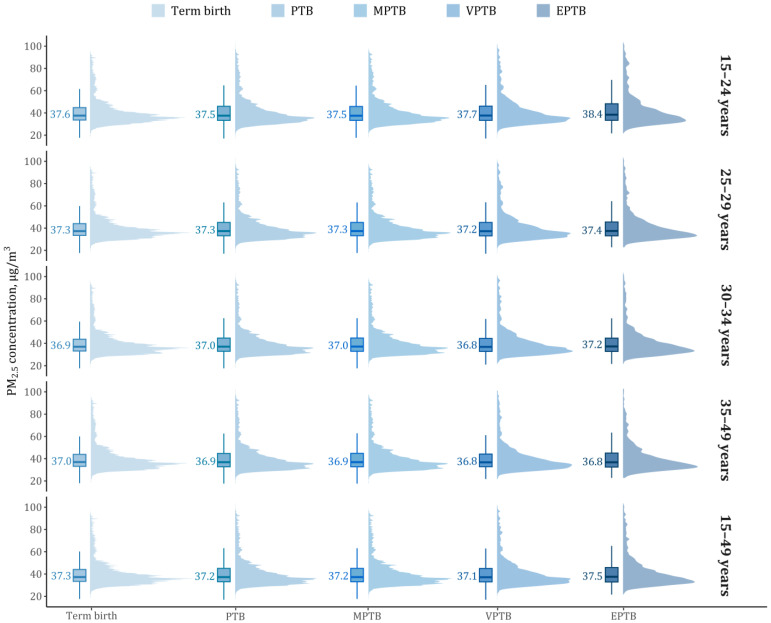
Boxplots and kernel density curves of age-specific PM_2.5_ exposure in term births and PTB outcomes. Abbreviations: PTB, preterm birth; MPTB, moderate to late preterm birth; VPTB, very preterm birth; EPTB, extremely preterm birth; PM_2.5_, fine particulate matter.

Table 1 describes the characteristics of the population enrolled in this study stratified by maternal age groups. All singleton live births (*n* = 3,839,531) had an average GA of 38.5 (SD = 1.7) weeks, and about a half (51.6%) were male babies. During 2013–2018, we recorded a total of 246,055 (6.4%) PTB newborns, including 215,624 (5.6%) MPTB, 22,031 (0.6%) VPTB, and 8400 (0.2%) EPTB, respectively. Nearly half of the mothers (47.5%) were educated below high school level, and over three-quarters (76.6%) resided in the city. The mean PM_2.5_ concentration was 41.2 μg/m^3^ (SD = 12.7) throughout the entire pregnancy, and slightly lower estimates were seen in the first trimester (40.8 μg/m^3^, SD = 14.5) than the second trimester (41.1 μg/m^3^, SD = 14.9) and the third trimester (41.7 μg/m^3^, SD = 14.5). Among all mothers, PM_2.5_ exposure levels showed a negatively skewed distribution, with comparable median exposure levels ranging between 36.8 and 38.4 μg/m^3^ (Figure 1). Compared with mothers with term births, those with EPTB were generally exposed to higher PM_2.5_ levels, notably among the adolescent mothers (15–24 years).

**Figure 2:**
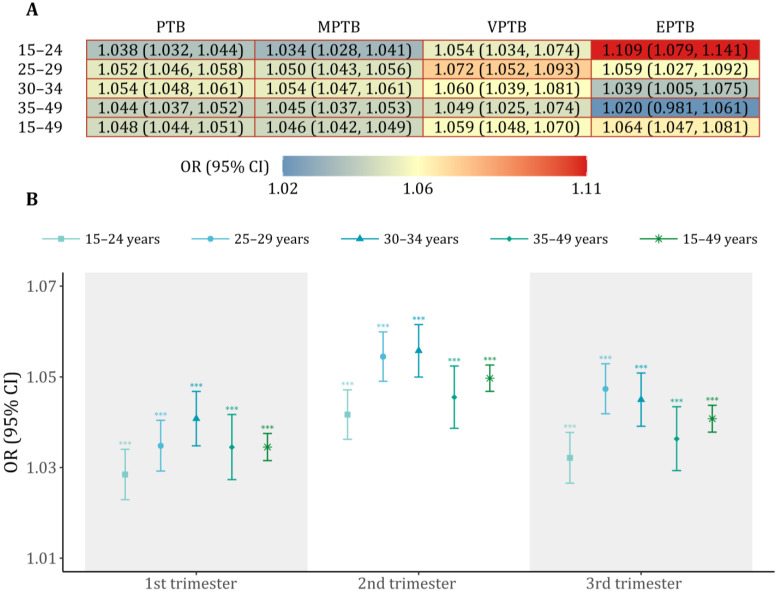
Age-specific odds ratio of PTB outcomes associated with a 10 μg/m^3^ increase in PM_2.5_ exposure throughout the entire pregnancy (**A**) and each trimester (**B**). Note: *** indicates statistical significance. Abbreviations: OR, odds ratio; CI, confidence interval; PTB, preterm birth; MPTB, moderate to late preterm birth; VPTB, very preterm birth; EPTB, extremely preterm birth.

Figure 2 shows the associations between PM_2.5_ exposure and PTB outcomes over the entire pregnancy and different trimesters. Broadly, positive effects of PM_2.5_ exposure on increased PTB risks were consistently observed across pregnancy trimesters. For each 10 μg/m^3^ increase in PM_2.5_ over the entire pregnancy, the estimated odds were 1.048 (95% CI: 1.044, 1.051) for overall PTB, 1.046 (95% CI: 1.042, 1.049) for MPTB, 1.059 (95% CI: 1.048, 1.070) for VPTB, and 1.064 (95% CI: 1.047, 1.081) for EPTB, respectively. Among the PTB subtypes, VPTB and EPTB were more strongly associated with PM_2.5_ exposure, particularly among younger mothers (15–29 years). In age-specific analyses, exposure-related risk of PTB in the reproductive age (25–34 years) group was estimated to be modestly greater than that in the adolescent pregnancy (15–24 years) and older pregnancy (35–49 years) groups. We observed similar trends across different gestational stages, while more pronounced risk effects were seen during the middle stage of pregnancy in all age subgroups. Sensitivity analyses additionally adjusted for area-level socioeconomic indicators demonstrated a minimal variation in the estimated associations between PM_2.5_ exposure and PTB subtypes across different model specifications (Table 2).

**Figure 3:**
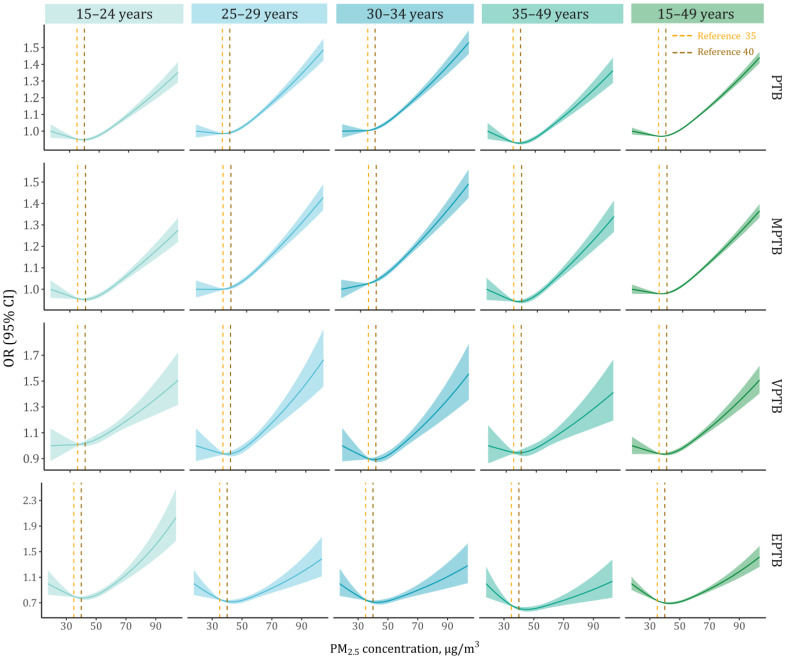
Exposure–response associations of PM_2.5_ exposure during the entire pregnancy with various PTB outcomes, stratified by maternal age. The vertical dashed lines indicate reference PM_2.5_ concentrations of 35 μg/m^3^ and 40 μg/m^3^. Abbreviations: OR, odds ratio; CI, confidence interval; PM_2.5_, fine particulate matter; PTB, preterm birth; MPTB, moderate to late preterm birth; VPTB, very preterm birth; EPTB, extremely preterm birth.

Figure 3 shows age-stratified E-R associations of PM_2.5_ exposure with overall PTB and its subcategories. We observed generally parallel evidence for J-shaped associations between PM_2.5_ exposure during the entire pregnancy and the risks of various PTB outcomes. Overall, the E-R curves indicated a potential threshold estimated to be approximately 40 μg/m^3^, above which PTB risk was markedly elevated. Specifically, at higher exposure concentrations, notably steeper slopes of PM_2.5_-related risks were seen among mothers aged 25–34 years for MPTB but among mothers aged 15–24 years for EPTB. Compared with MPTB, both EPTB and VPTB showed much broader confidence bands in the estimated exposure–risk curves.

**Figure 4:**
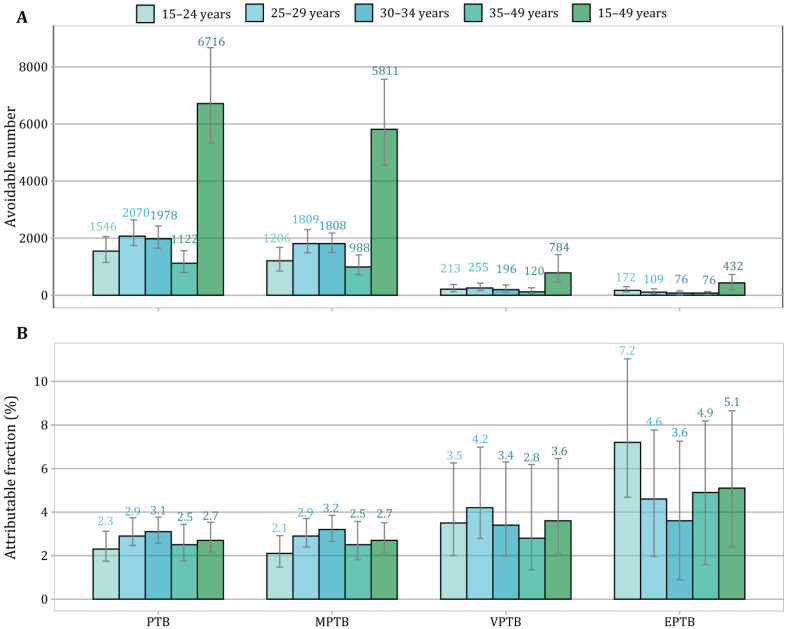
Estimates of avoidable PTB burden by achieving WHO’s IT1 for annual PM_2.5_ concentration in Iran during 2013–2018: (**A**) estimated number of avoidable cases; (**B**) attributable fraction. Abbreviations: PTB, preterm birth; MPTB, moderate to late preterm birth; VPTB, very preterm birth; EPTB, extremely preterm birth; PM_2.5_, fine particulate matter; WHO, World Health Organization; IT, interim target.

Figure 4 calculates PTB burden attributable to maternal PM_2.5_ exposure exceeding WHO’s IT1 (35 μg/m^3^) in Iran. Assuming causality, meeting the IT1 for gestational PM_2.5_ exposure could potentially avoid a total of 6716 (95% CI: 5336, 8678) preterm infants during the study period 2013–2018, nearly accounting for 2.7% (95% CI: 2.2%, 3.5%) of the observed PTB cases in Iran. In comparative analyses for PTB subcategories, the greatest avoidable fraction was estimated for EPTB (5.1%, 95% CI: 2.4%, 8.6%), followed by VPTB (3.6%, 95% CI: 2.1%, 6.5%) and MPTB (2.7%, 95% CI: 2.1%, 3.5%). For overall PTB, MPTB, and VPTB, we observed greater PM_2.5_-attributable fractions among mothers aged 25 to 34 years; however, we estimated a considerably higher fraction of 7.2% (95% CI: 4.7%, 11.0%) for EPTB among the adolescent mothers (15–24 years).

## 4. Discussion

To the best of our knowledge, this is the first nationwide cohort analysis to examine the association between PTB outcomes and PM_2.5_ exposure during pregnancy in Middle Eastern countries. By investigating approximately 3.8 million singleton live births, we found that PM_2.5_ exposure during pregnancy was associated with increased risks of PTB outcomes. Stratified analyses indicated considerably heightened PM_2.5_-related risks among mothers aged 25–34 years and in the second trimester. We detected J-shaped exposure–risk curves, and counterfactual analysis highlighted that improved air quality may greatly mitigate the PTB burden in Iran.

For overall PTB, we estimated an OR of 1.048 (95% CI: 1.044, 1.051) for a 10 μg/m^3^ increase in prenatal PM_2.5_ exposure. Consistent with a large body of population-based evidence [12], our results revealed that PM_2.5_ exposure during pregnancy was associated with increased risks of different PTB outcomes. Maternal PM_2.5_ exposure may elevate PTB risk through multiple biological pathways. Inhaled PM_2.5_ can induce systemic and placental inflammation and oxidative stress, impairing placental [29]. PM_2.5_ has been linked to disturbances in uterine and umbilical–placental blood flow, premature placental aging, and endocrine disruption, all of which may contribute to shortened gestation [30]. Recent findings also suggest that PM_2.5_ may alter placental mitochondrial function and epigenetic regulation [31,32]. Despite the existing positive associations between PM_2.5_ and elevated risk of PTB, the observed effects varied considerably between populations and by exposure levels. For each 10 μg/m^3^ rise in PM_2.5_ exposure, the risk of overall PTB was increased by 11% (95% CI: 9%, 14%) in North America [33] and 34% (95% CI: 2%, 76%) in Europe [34]. In locations with higher-level pollution, populations seemed to be at lower vulnerability. For instance, multi-country investigations in Africa [35] estimated an 8% (95% CI: 1%, 16%) higher PM_2.5_-related PTB risk for an interquartile range (33.9 μg/m^3^) rise in exposure, and a largely comparable effect size of 7% (95% CI: 7%, 8%) for each 29 μg/m^3^ rise was reported in a nationwide birth cohort study in 336 Chinese cities [36]. In line with previous studies [37,38], we observed stronger associations of PM_2.5_ exposure with VPTB and EPTB in the Iranian population. Notably, adolescent mothers (15–24 years) exhibited heightened vulnerability to PM_2.5_-related EPTB, potentially due to their greater propensity for being exposed to polluted air and relatively lower socio-economic status. Given the paucity of national population-based evidence from the Middle East, our study may potentially fill in some knowledge gaps and provide valuable insights into global air quality health disparities. This contribution is particularly novel given the arid climate and dust-heavy environment of the region, which differ from high-income countries [11].

Only a limited number of prior studies have depicted the nonlinear E-R functions between PM_2.5_ exposure and various PTB outcomes. In our E-R analyses among the Iranian population, we observed a sharply elevated PM_2.5_-related risk when PM_2.5_ concentrations exceeded approximately 40 μg/m^3^ (nearly J-shaped), indicating a potential threshold for the effect of PM_2.5_ exposure during pregnancy on PTB. One possible explanation is that low PM_2.5_ levels may trigger mild stress, activating adaptive mechanisms at the maternal–placental interface, while higher levels may cause placental dysfunction, vascular injury, and inflammation, increasing PTB risk [30]. Similarly, several birth cohort studies across China [18,39] and the U.S. [40] observed potential thresholds that may vary with population exposure levels. More evidence-based efforts in identifying air pollution exposure thresholds could have public health implications for guiding the development of revised air quality standards and targeted interventional actions (e.g., limit outdoor activities or use air filtration systems) [41]. E-R curves derived from our study reinforce the evidence that linear risk extrapolation, especially in heavily polluted settings, may potentially underestimate PM_2.5_-attributable PTB burden; however, more sophisticated analyses under the causal inference framework are still warranted to validate our findings [21].

Based on the E-R functions derived in this national analysis, we estimated that 2.7% (95% CI: 2.2%, 3.5%) of PTB cases during 2013–2018 could be avoided in Iran by achieving the predetermined WHO’s IT1 for ambient PM_2.5_. While this level is higher than Iran’s current annual PM_2.5_ guideline of 12 μg/m^3^, our findings still highlight the potential health benefits of further exposure reduction, particularly for high-burden subgroups. PTB burden attributable to maternal PM_2.5_ exposure has been quantified in prior counterfactual analyses, globally or regionally, on the basis of diverse methodologies [42,43,44,45]. For the year 2010 alone, a total of 2.7 (95% CI: 1.8, 3.5) million PTB infants were attributed to PM_2.5_ exposure exceeding 10 μg/m^3^, accounting for 18% (95% CI: 12%, 24%) of PTB cases globally [44]. In the American population, an estimated 3.32% of national PTB cases (corresponding to 15,808 PTBs) could be averted if ambient PM_2.5_ concentration declined to 8.8 μg/m^3^ in 2010 [45]. Both of these two studies relied on linear estimates of PM_2.5_-related PTB risk obtained from meta-analyses and may greatly oversimplify the underlying exposure–risk relationship and underestimate the attributable burden. Despite that MPTB accounted for the majority of PM_2.5_-attributable PTB cases in our counterfactual analyses by achieving WHO’s IT1, the AFs estimated a significantly greater proportion of avoidable VPTB and EPTB burden (Figure 4). Compared to MPTB cases, children born with EPTB and VPTB may experience more severe health outcomes in later life, including higher risks of mortality and morbidity along with short- and long-term consequences [46]. Our findings emphasize that a higher proportion of VPTB and EPTB infants could be avoided by mitigating PM_2.5_ pollution, providing a unique perspective on the health benefits of improving air quality, particularly in heavily polluted LMICs [5]. This highlights an opportunity for policy action. In the context of Iran, this may involve prioritizing emission reductions in high-burden provinces, integrating air pollution awareness into prenatal care services, and enhancing national air quality monitoring to support both public health and environmental policy.

Stratified analyses revealed heterogeneity across age groups, showing greater susceptibility to PM_2.5_-related risks of overall PTB among women aged 25–34 years. Evidence from Africa [35] and China [47] reported stronger associations between PM_2.5_ and PTB among individuals aged over 30 years. This inconsistency may be partially explained by the between-study heterogeneity in demographic characteristics, and more sophisticated and cross-population investigations are expected to further elucidate the moderate role of maternal age factors. Distinguishing critical time windows can also facilitate the targeted prevention of PTB, while evidence remains inconclusive [7]. Paralleling with a meta-analysis involving sixty international studies [48], our study also detected a relatively stronger effect of PM_2.5_ exposure on PTB during the second trimester (14–26 weeks of gestation). Also, two prior analyses focusing on the relationship of weekly PM_2.5_ exposure with PTB risk identified exposure windows of susceptibility in 17–24 weeks [33] and 20–28 weeks [49] of the gestational period. Conversely, the third trimester was identified as a sensitive exposure window of maternal PM_2.5_ exposure and PTB in two nationwide birth cohorts in Chinese populations [10,50]. These findings highlight the need for further comprehensive insights into the roles of methodological approach and population demography when investigating the potential windows of maternal PM_2.5_ exposure. By identifying vulnerable groups and delineating critical gestational periods, our findings provide local evidence to guide tailored interventions and inform public health strategies. Building on this approach, future studies could explore specific patterns at the subnational level to capture potential regional differences and inform more geographically tailored public health strategies in Iran.

This study has several limitations. First, although several covariates were included in the analysis, residual confounding due to unmeasured factors such as maternal smoking, alcohol use, nutrition, and indoor environmental exposures cannot be excluded [51]. To enhance confounding control and exposure assessment, incorporating more granular individual-level data on socioeconomic status, lifestyles, and indoor environmental exposure would be beneficial to strengthen causal inference in future research. Second, the exposure assessment is subject to potential misclassification, as PM_2.5_ concentrations were assigned based on the delivery hospital. Future studies incorporating individual-level residential data, higher-resolution exposure estimates, or personal monitoring based on smart wearable devices are warranted to improve spatial precision and reduce potential bias in exposure assessment. Third, although meteorological factors and co-pollutants were not explicitly included in the model, we adjusted for season of birth and residential area to partially account for temporal and spatial variations in environmental exposures.

## 5. Conclusions

In summary, our national cohort analysis of over 3.8 million singleton live births provided unique evidence for increased risk of various PTB outcomes associated with PM_2.5_ exposure during pregnancy in Iran, particularly among mothers aged 25–34 and during the second trimester. A J-shaped exposure–response relationship suggested a potential threshold around 40 μg/m^3^. Counterfactual analyses revealed higher avoidable burdens for VPTB and EPTB in Iran. Our findings should contribute to the discourse on environmental justice and health inequality, emphasizing the urgent need for global efforts to deal with the threat of air pollution and uphold maternal and infant rights to healthy development, particularly in high-pollution nations.

## Figures and Tables

**Table 1:** Summary characteristics of the population stratified by maternal age groups.

Characteristics	15–24 Years (*n* = 1,112,264)	25–29 Years (*n* = 1,207,306)	30–34 Years (*n* = 969,491)	35–49 Years (*n* = 550,470)	15–49 Years (*n* = 3,839,531)
Fetal information, *n* (%)					
PTB	65,873 (5.9)	70,550 (5.8)	64,333 (6.6)	45,299 (8.2)	246,055 (6.4)
MPTB	57,449 (5.2)	62,134 (5.1)	56,519 (5.8)	39,522 (7.2)	215,624 (5.6)
VPTB	6034 (0.5)	6063 (0.5)	5718 (0.6)	4216 (0.8)	22,031 (0.6)
EPTB	2390 (0.2)	2353 (0.2)	2096 (0.2)	1561 (0.3)	8400 (0.2)
GA (week), mean (SD)	38.7 (1.6)	38.6 (1.6)	38.4 (1.6)	38.3 (1.7)	38.5 (1.6)
Male sex	572,789 (51.5)	622,174 (51.5)	500,741 (51.6)	285,058 (51.8)	1,980,762 (51.6)
Season of birth					
Spring	244,769 (22.0)	259,654 (21.5)	205,911 (21.2)	119,781 (21.8)	830,115 (21.6)
Summer	276,764 (24.9)	303,758 (25.2)	237,333 (24.5)	131,831 (23.9)	949,686 (24.7)
Fall	322,125 (29.0)	354,198 (29.3)	288,285 (29.7)	161,275 (29.3)	1,125,883 (29.3)
Winter	268,606 (24.1)	289,696 (24.0)	237,962 (24.5)	137,583 (25.0)	933,847 (24.3)
Cesarean section	425,461 (38.3)	621,794 (51.5)	565,792 (58.4)	328,091 (59.6)	1,941,138 (50.6)
Maternal characteristics, *n* (%)					
Education attainment					
Below high school	575,172 (51.7)	514,211 (42.6)	423,149 (43.6)	309,704 (56.3)	1,822,236 (47.5)
High school	394,381 (35.5)	379,139 (31.4)	280,765 (29.0)	121,277 (22.0)	1,175,562 (30.6)
College and above	142,711 (12.8)	313,956 (26.0)	265,577 (27.4)	119,489 (21.7)	841,733 (21.9)
City residence	792,323 (71.2)	947,613 (78.5)	778,635 (80.3)	421,242 (76.5)	2,939,813 (76.6)
Iranian nationality	1,050,770 (94.5)	1,167,419 (96.7)	942,983 (97.3)	534,466 (97.1)	3,695,638 (96.3)
MIP (%), mean (SD)	43.2 (11.2)	44.6 (11.1)	45.1 (11.1)	44.2 (11.2)	44.3 (11.2)
UR (%), mean (SD)	10.7 (2.5)	10.7 (2.5)	10.7 (2.5)	10.7 (2.5)	10.7 (2.5)
Diabetes	11,752 (1.1)	24,735 (2.0)	32,116 (3.3)	27,935 (5.1)	96,538 (2.5)
Hypertension	10,971 (1.0)	14,067 (1.2)	14,746 (1.5)	13,887 (2.5)	53,671 (1.4)
Primipara	768,776 (69.1)	502,512 (41.6)	239,292 (24.7)	75,397 (13.7)	1,585,977 (41.3)
History of abortion	107,761 (9.7)	185,410 (15.4)	199,887 (20.6)	149,625 (27.2)	642,683 (16.7)
PM_2.5_ (μg/m^3^), mean (SD)					
Trimester 1	41.3 (15.0)	40.8 (14.4)	40.3 (14.2)	40.5 (14.3)	40.8 (14.5)
Trimester 2	41.6 (15.2)	41.1 (14.8)	40.7 (14.7)	41.0 (15.0)	41.1 (14.9)
Trimester 3	42.1 (14.8)	41.7 (14.4)	41.3 (14.3)	41.4 (14.4)	41.7 (14.5)
Entire pregnancy	41.7 (13.0)	41.2 (12.7)	40.8 (12.5)	41.0 (12.7)	41.2 (12.7)

Note: The sum of percentages from multiple subgroups may not equal 100% exactly due to the use of the rounding-off method. Abbreviations: PTB, preterm birth; MPTB, moderate to late preterm birth; VPTB, very preterm birth; EPTB, extremely preterm birth; GA, gestational age; MIP, medical insurance participation; UR, unemployment rate; SD, standard deviation; PM_2.5_, fine particulate matter.

**Table 2:** Sensitivity analyses for associations between PM_2.5_ exposure and PTB subtypes.

Model	OR (95% CI)	*p*-Value for Effect Difference
PTB		
Main analysis	1.048 (1.044, 1.051)	Reference group
Main analysis + MIP	1.047 (1.044, 1.051)	0.891
Main analysis + UR	1.048 (1.044, 1.051)	0.985
Main analysis + MIP + UR	1.047 (1.044, 1.050)	0.838
MPTB		
Main analysis	1.046 (1.042, 1.049)	Reference group
Main analysis + MIP	1.045 (1.041, 1.048)	0.680
Main analysis + UR	1.046 (1.042, 1.049)	0.963
Main analysis + MIP + UR	1.044 (1.041, 1.048)	0.549
VPTB		
Main analysis	1.059 (1.048, 1.070)	Reference group
Main analysis + MIP	1.066 (1.055, 1.077)	0.356
Main analysis + UR	1.060 (1.049, 1.070)	0.927
Main analysis + MIP + UR	1.069 (1.058, 1.080)	0.192
EPTB		
Main analysis	1.064 (1.047, 1.081)	Reference group
Main analysis + MIP	1.064 (1.047, 1.082)	0.952
Main analysis + UR	1.064 (1.047, 1.081)	0.986
Main analysis + MIP + UR	1.065 (1.048, 1.082)	0.918

Abbreviations: PM_2.5_, fine particulate matter; PTB, preterm birth; MPTB, moderate to late preterm birth; VPTB, very preterm birth; EPTB, extremely preterm birth; OR, odds ratio; CI, confidence interval; MIP, medical insurance participation; UR, unemployment rate.

## Data Availability

The data that support the findings of this study are derived from the Iranian national birth registry. Due to ethical and legal restrictions related to the use of sensitive health information, these data are not publicly available. Access to the data may be granted upon reasonable request and with permission from the relevant authorities.
